# A dual adaptive watermarking scheme in contourlet domain for DICOM images

**DOI:** 10.1186/1475-925X-10-53

**Published:** 2011-06-17

**Authors:** Farhad Rahimi, Hossein Rabbani

**Affiliations:** 1 Department of Biomedical Engineering, Isfahan University of Medical Sciences, Isfahan, Iran; 2 Medical Image and Signal Processing Research Center, Isfahan University of Medical Sciences, Isfahan, Iran

## Abstract

**Background:**

Nowadays, medical imaging equipments produce digital form of medical images. In a modern health care environment, new systems such as PACS (picture archiving and communication systems), use the digital form of medical image too. The digital form of medical images has lots of advantages over its analog form such as ease in storage and transmission. Medical images in digital form must be stored in a secured environment to preserve patient privacy. It is also important to detect modifications on the image. These objectives are obtained by watermarking in medical image.

**Methods:**

In this paper, we present a dual and oblivious (blind) watermarking scheme in the contourlet domain. Because of importance of ROI (region of interest) in interpretation by medical doctors rather than RONI (region of non-interest), we propose an adaptive dual watermarking scheme with different embedding strength in ROI and RONI. We embed watermark bits in singular value vectors of the embedded blocks within lowpass subband in contourlet domain.

**Results:**

The values of PSNR (peak signal-to-noise ratio) and SSIM (structural similarity measure) index of ROI for proposed DICOM (digital imaging and communications in medicine) images in this paper are respectively larger than 64 and 0.997. These values confirm that our algorithm has good transparency. Because of different embedding strength, BER (bit error rate) values of signature watermark are less than BER values of caption watermark. Our results show that watermarked images in contourlet domain have greater robustness against attacks than wavelet domain. In addition, the qualitative analysis of our method shows it has good invisibility.

**Conclusions:**

The proposed contourlet-based watermarking algorithm in this paper uses an automatically selection for ROI and embeds the watermark in the singular values of contourlet subbands that makes the algorithm more efficient, and robust against noise attacks than other transform domains. The embedded watermark bits can be extracted without the original image, the proposed method has high PSNR and SSIM, and the watermarked image has high transparency and can still conform to the DICOM format.

## Background

In the recent years, medical images are produced from a wide variety of digital imaging equipments, such as computed tomography (CT), magnetic resonance imaging (MRI), computed radiography (CR) and so forth. With the increasing use of internet and appearance of new system such as picture archiving and communication systems (PACS), the usability of digital form of medical images has been increased [1]. Images in digital imaging equipments can be printed on films or papers. Moreover, in these equipments images with patient data in DICOM format can be stored on different types of storage media such as CD or DVD [2]. DICOM is a standard file format for transmission and storage of digital medical images in health care centers [3]. Header in DICOM image format stores patient's information such as patient identification number, name, sex, and age [4]. Insurance companies, hospitals and patients may want to change this data for various reasons. Therefore, protecting medical images against this threat is necessary. Watermarking can be used as a solution. Digital image watermarking means placing a hidden data (patients information) within the body of an image without changing image size or format. After embedding the data, watermarked medical image can still conform to the DICOM format [5].

From the literature, the purposes of medical image watermarking are classified into two categories [6]:

1- Tamper detection

2- Hiding EPR (electronic patient records) for confidentiality and authentication.

Tamper detection in watermarking are used to locate the regions or pixels of the image where tampering has been done. Confidentiality means that only the eligible users have access to the information. Authentication intends that the information belongs indeed to the correct patient and is issued from the correct source. In digital imaging equipment, authentication is obtained via embedding patient's information in images. When patient's information is extracted in health care centers, it can be used to prove ownership.

Depending on the purpose of the watermarking (tamper detection or hiding patient's information), a proper watermarking technique is chosen accordingly.

The digital watermark should be hidden in the image. However, it generally introduces some amount of imperceptible distortion in the image. For medical images, there is a region that is important for diagnosis, and this region should not be altered. This important area is called ROI (region of interest) [7]. Because embedding data in medical images must not cause any visual artifacts in ROI that may affect the interpretation by medical doctors, watermarking in RONI (region of non-interest) can be used in medical image watermarking process.

In order to enhance confidentiality and authentication, in this paper, we use a dual watermarking scheme. We focus on two kinds of watermark hiding. In *caption watermarking*, by hiding patient's information in ROI, both authentication and confidentiality are achieved and gives a permanent link between the patient and the medical data. In *signature watermarking*, we hide the physician's digital signature or identification code in RONI for the purpose of origin authentication.

To achieve better performance in terms of perceptually, invisibility and robustness, we use adaptive quantization parameters for data hiding. Because the energy distribution is an important characteristic for digital image processing [8], we use a model that employs this parameter for determining the adaptive quantization parameter. The embedding strength is more or less proportional to the value of energy to have better robustness and transparency in proposed method.

Block with large value of energy contains big coefficients and should be treated as a significant block in comparison with other blocks. The watermark bit must be embedded into this significant block with the larger quantization parameters to improve the robustness.

In the following, before allocating sections for methods, results and conclusion, a short review of medical images' watermarking techniques is presented, and then the contourlet transform is introduced briefly in order to understand the proposed method and results better.

## A short review on watermarking techniques

For various standpoints, current watermarking techniques can be categorized to different classes. From embedding location standpoint, there are two main classes [9]. The first comprises the spatial domain methods, which embed the watermark by directly modifying the pixel values of the original image. The second class includes the transform domain methods, which embed the data by changing the transform domain image coefficients. Embedding watermark in the frequency domain can provide more robustness watermarking than spatial domain. From another standpoint three kinds of watermarking methods were identified for medical images [10]. The first class includes methods that embed information within RONI in order not to threat the diagnosis capability. The second class comprises reversible watermarking methods. A reversible watermarking scheme involves embedding a watermark into the original image in an invertible manner in that when the watermark is extracted, the original image can be recovered completely. The third class includes classical watermarking methods that embed information within image in spatial domain or transform domain, in order to minimize the distortion.

Robust and fragile watermarks are the two wide categories of the watermarks. Robust watermarking is chiefly intended towards copyright protection. On the contrary fragile watermarking is built to identify any minute alternation to the original digital content.

Moreover, Non-Blind, Semi-Blind and Blind methods are the divisions of watermarking. In case of Non-Blind methods, the original image are employed for the extraction of watermark, while Semi-Blind techniques demand the presence of the watermark bit sequence, whereas the detection process in the Blind methods do not require the original image.

In the following, some suggested methods in two domains (spatial and frequency) are reviewed.

### Spatial domain techniques

*Nayak et al *[11] proposed a method for compact storage and transmission of patient's information with medical images. They have used a reversible watermarking technique to hide the patient's information (text data) within the retinal fundus image. Before embedding, for high safety they have encrypted patient's information with error control coding (ECC) such as Hamming, BCH, and RS codes.

In the method suggested by *Trichili et al *[12], patient data (170 characters) are converted to binary data and a private key scrambles it. For more security the scrambled data are encrypted. From original image, the virtual border creates by mirror effect. The encrypted data was inserted in the less significant bits of the new border. This technique dose not influences the quality of the original image. At the reception, inverse procedure is done.

*Zian et al *[13] proposed a reversible technique where the original image can be recovered completely. They specify ROI and RONI in ultrasound images and after that the calculated Hash code of ROI is embedded into the least significant bits (LSB) of RONI. Because the original values of RONI are zero before embedding, at the receiver end the watermark is extracted from LSB's of RONI and those pixels are reset back to zero.

*Osamah et al *[6] proposed a fragile watermarking scheme that is a combination of two reversible techniques based on different expansions for patient's information hiding and protecting ROI with tamper assessment and retrieval capability. Patient's data are embedded into ROI while the average of blocks inside ROI for recovering data, are embedded into RONI.

For obtaining a robust watermarking scheme versus active attacks such as geometrical attacks, *Raul et al *[14] applied image moment theory for watermarking of medical images. In order to reduce the amount of data to embed, they compressed the DICOM metadata. For more security, the compressed data were encrypted. The centroid of the image can be calculated from two first order moments. Embedding watermark is done in areas with low homogeneity, which can be acquired by scanning the image in a spiral way using the centroid as the origin of this scan. For extraction, the image is scanned in the same spiral way starting from the centroid of the image. By comparing the grayscale level of the center pixel of an area with the grayscale level of its mean, one bit of the watermark is extracted from the area.

### Frequency Domain Techniques

*Dandapat et al *[15] proposed a wavelet-based data embedding technique for embedding medical data such as patient's information and patient's signal into medical images. They used diagnostic distortion measure (DDM) to evaluate and separate the wavelet coefficient into two sets, diagnostically least sensitive coefficient and diagnostically sensitive coefficient. The patient's data are embedded into sets of diagnostically least sensitive coefficient by using LSB coding.

For authentication and confidentiality of both origin and data in medical images, the multiple watermarking was proposed by *Giakuomaki et al *[16]. They have used Haar discrete wavelet transform combined with a suitable quantization method. The scheme embeds a robust watermarking containing the doctor's digital signature for source authentication, and caption watermark conveying patient's information, health history, and fragile watermark for tamper detection. They then augmented their technique gradually to increase its robustness and security [17-20].

## Contourlet transform

**Figure 1:**
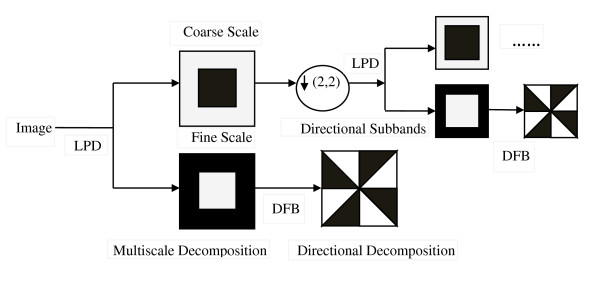
**Contourlet decomposition framework**.

**Figure 2:**
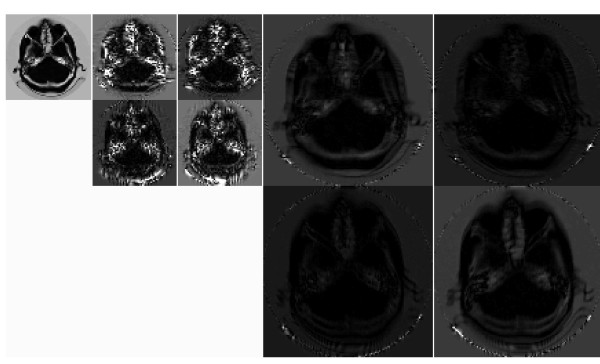
**The contourlet transform of a CT brain image**.

The contourlet transform [21], as introduced by Minh Do and Martin Vetterli, is a new image decomposition scheme, which provides a flexible multiresolution representation for two dimensional signals. It makes use of the Laplacian pyramid decomposition (LPD) for the multiresolution representation of the image. In the contourlet transform, the Laplacian pyramid decomposes an image into a low frequency subband and a high frequency subband. After this, a directional decomposition is performed on every band-pass image using directional filter banks (DFB). The contourlet transform is unequaled since the number of directional bands could be indicated by the user at any resolution. Finally, the image is represented as a set of directional subbands at multiple scales. Discrete countourlet transform is able to capture the directional edges and contours superior to discrete wavelet transform. The schematics structure of contourlet transform [22] and an example of contourlet decomposition of a CT brain image are illustrated in Figure 1 and 2 respectively.

## Methods

**Figure 3:**
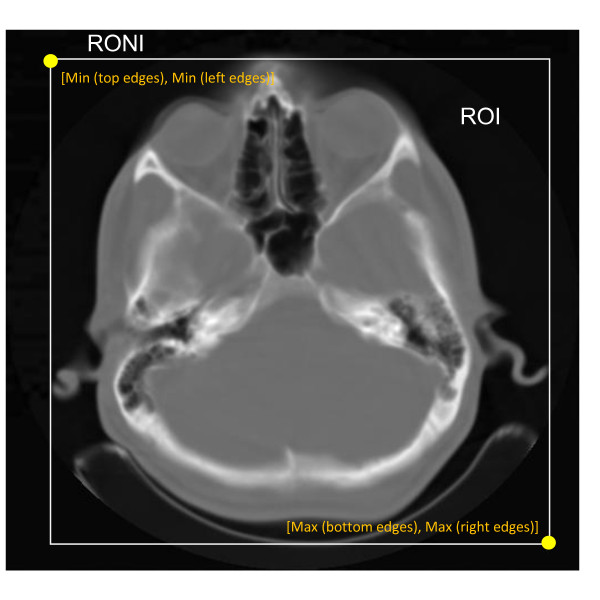
**A proposed test image and automatically selection of ROI and RONI**.

The proposed contourlet-based blind adaptive watermarking scheme is described in this section. Although there exist several researches about watermarking using contourlet transform [8,22-40], the novelty of our paper can be described as follows. In our paper we use an automatically selection for ROI. To achieve better performance in terms of perceptually, invisibility and robustness, we use adaptive quantization parameters for data hiding. Because the energy distribution is an important characteristic for digital image processing, we use a model that employs this parameter for determining the adaptive quantization parameter. Because of importance of ROI in interpretation by medical doctors rather than RONI, we propose an adaptive dual watermarking scheme with different embedding strength in ROI and RONI. This work causes our method has high transparency. In addition, in the most previously published methods, DICOM image change to grayscale image but in our method watermarked image can still conform to the DICOM format. Suppose the original image is I_0 _that should be decomposed in contourlet domain. Before embedding process, the following preprocessing steps are done as follows:

1. In this paper we use an automatically selection for ROI. Unlike popular definition for RONI (any area of the image that doesn't contain any clinical information), we defined RONI as the region of background (black area inside an image). For each row of the image, the left and the right edges of the image are recorded, similarly for each column of the image, the top and bottom edges of the image are recorded too. For an image of dimensions M × N, the left and the right edges of the image form two vectors L and R of size M, and the upper and lower edge of the image construct two vectors T and B of size N. For each vector, we select l = min(L), r = max(R), t = min(T) and b = max(B), and then we define a rectangle of which the left upper corner has coordinates (t, l) and the bottom right one is (b, r). In the proposed method, a rectangle is automatically selected for ROI (Figure 3).

2. Watermark is reshaped to binary vector (W={w_1_, w_2_, w_3_,..., w_K_}, w_k _∈ {0,1}).

3. In view of the robustness, we choose I_L_, lowpass subband of decomposed I_0_, for embedding and W is embedded into I_L _in contourlet domain. For more invisibility the embed process can be done in the detail subbands.

### Watermark Embedding Process

I_L _is divided into non-overlapping blocks A_i _of size b × b, i = 1,2,..., M, where M is the number of the blocks.

The energy value of each block A_i _is computed according to(1)

For each block A_i _the adaptive quantization step value δ_i _is computed as follows.(2)

where δ_0 _is the basic quantization step that is different in ROI and RONI and served as a secret key, and the function floor represents the round-off operation.

Using singular value decomposition (SVD), similar to proposed embedding procedure in [40], the singular value vectors of each block A_i _are computed. SVD is a mathematical tool used to analyze matrices. In SVD, a matrix is decomposed into three matrices of same size (i.e.,  where U's columns are basis vectors of A_i_^T^A_i_, V's columns are basis vectors of A_i_A_i_^T^, and the diagonal values in diagonal matrix S_i _= diag(γ_i1_, γ_i2_,..., γ_iw_) are singular values of A_i_).

By the singular values of each block  is computed (where ||·|| represents the Euclidean norm) and quantized by adaptive quantization step δ_i _that represents the quantization level as follows:(3)

 is obtained according to the value of w_i_, and N_i _is obtained using the following formula:(4)

The above equation means that we modify N_i _according to the value of w_i_, and N_i _as follows. If (w_i _= 1 and N_i _is an odd number) or (w_i_= 0 and N_i _is an even number), then we change the value of N_i_.

Finally, using the value , the modified singular values are computed as follows:(5)

**Figure 4:**
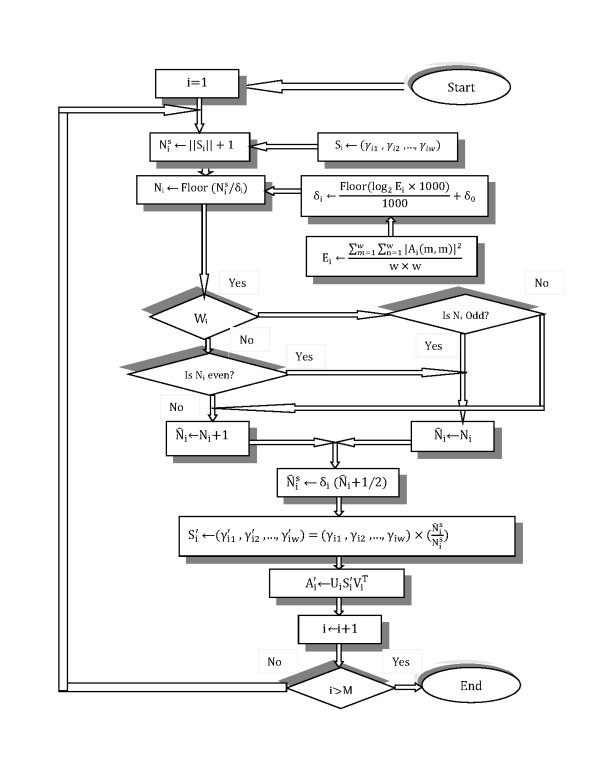
**The flowchart of "Watermark Embedding Process"**.

Using the modified singular values, watermarked block  is obtained . By inverse transform, the watermarked image I_0_' is reconstructed from all watermarked blocks. The flowchart of "Watermark Embedding Process" is shown in Figure 4.

### Watermark Extraction Process

For watermark extraction, our proposed method only requires the size of binary vector (W), and basic quantization step (δ_0_) (it doesn't require the original image or any of its characteristics).

Similar embedding process, the watermarked image I_0_' is converted to the contourlet domain and lowpass subband I_L_' is selected for extraction. The extracting procedure is given as follows:

At first I_L_' is divided into non-overlapped blocks  of size b × b, i = 1,2,..., M where M is the number of the blocks.

Then,  is computed and quantized by adaptive quantization step δ_i _that is computed alike embedding process  denotes a diagonal metric formed by the singular values of each block ).

 is calculated using the following equation.(6)

Finally, the embedded binary information  is extracted as follows.(7)

After two-level decomposition, the size of lowpass subband is 128 × 128 (the size of original image is 512 × 512). The size of blocks b is set to 2. The payload that can be archived by applying this technique in whole image (ROI and RONI) is . So, the maximum payload that can be achieved by this technique is 0.125 bpp.

Note that as a result of ROI and RONI definition with different size for each image, the payload that can be achieved by applying this technique is different. We selected minimum payload that can be achieved for all test images.

## Results

### Method evaluation

Unfortunately there is no standard method for automatically evaluating the amount of visible degradation of watermarked images, but in this paper we used two indicators for quantifying the similarity between original and watermarked images.

Peak Signal to Noise Ratio (PSNR) is used frequently as an objective image quality metric, but it does not consider characteristics of the human visual system (HVS) [41]. It is poor at comparing different watermarking methods, but provides a simple indicator for quantifying the similarity between original and watermarked images [41]. PSNR uses peak power of the original image and the mean squared value of the error signal. PSNR is expressed as follows:(8)

The second measure used in this paper is structural similarity measure (SSIM) index, which is a region-based numerical metric that places more emphasis on the HVS than PSNR. This metric is ideal for testing the similarities in medical images because it focuses on local rather than global image similarity [42]. Mathematically, for regions , it is expressed as(9)

SSIM compares the similarity in luminance (LC), contrast (CC), and structure (SC) of image regions for each pair of corresponding blocks. α, β, and λ are ≥ 1 and are used to weight the importance of each of the three components.

Luminance comparison is a function of corresponding blocks' mean intensity and is given by(10)

where  and  are the means of regions RI_0 _and  respectively, and c_1 _is a constant. Contrast comparison is a function of corresponding blocks' standard deviation and is expressed as(11)

where  and  are the standard deviations and  and  are the variances of regions RI_0 _and  respectively, and c_2 _is a constant.

Finally, the structural comparison is computed as the correlation coefficient of the two blocks and is given by(12)

where c_3 _is a constant and  is the correlation coefficient between regions RI_0 _and . We used above indicators for quantifying the similarity between original data and recovered data. We also used Bit Error Rate (BER) defined as bellow to evaluate the similarity between original EPR data and the recovered EPR data.(13)

where w_i _and  are the original and extracted EPR vectors respectively. In the lack of adverse attacks, BER was found to be zero.

The normalized correlation coefficient (NC) defined as bellow is also calculated to quantitatively analyze the likeness of the extracted watermark and the original watermark (logo) in *signature watermark*.(14)

where V(i, j), V'(i, j) are the original and extracted logos respectively, and M_1_, M_2 _are the size of logo image.

### Simulations

We use twenty brain CT images, taken from CT center of Isfahan Kashni Hospital and twenty brain MRI images, taken from MRI center of Isfahan Alzahra Hospital to test our watermarking procedure. The size of all images is 512 × 512. In addition our method could be performed using other types of medical images such as X-ray, Ultrasound, etc. Figure 3 demonstrates an example of these test images (and the defined ROI and RONI). As a result of ROI and RONI definition with different size for each image, the payload that can be achieved by applying this technique is different. We select the minimum payload that can be achieved for all test images.

**Figure 5:**
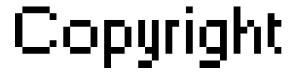
**Original logo**.

In our simulations, for *caption watermarking*, 230 characters of patient's information text are converted to ASCII cods, and then the ASCII cods are converted to binary vector. The *caption watermark *is embedded inside a rectangle in the ROI. We used one logo with size 10 × 40 (Figure 5) for *signature watermark *and it is embedded inside a rectangle in the RONI that surrounds the rectangular ROI area.

Images are transformed by contourlet transform using '9-7' pyramid filter and 'pkva' directional filter to obtain a two-level decomposition. The size of blocks b is set to 2 and for different embedding strength in ROI and RONI, the basic quantization step δ_0 _is not the same value. For ROI watermarking the basic quantization step is set to 6 and 4 for CT and MRI images respectively. Due to the fact that checker background of medical image may annoy the physician, the embedding strength of RONI has to be correctly selected. So, for RONI embedding, the basic quantization step achieved by lots of experiments is set 258 and 200 for CT and MRI images respectively. When we apply our method for medical images, the watermarked image can still conform to the DICOM format.

**Figure 6:**
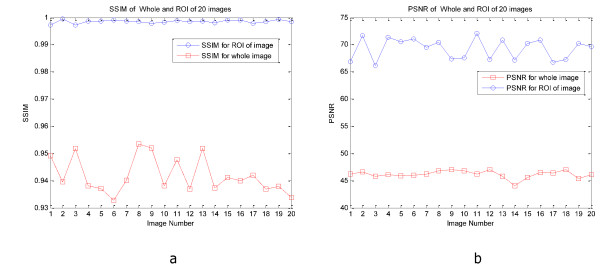
**PSNR and SSIM of whole and ROI of 20 CT images**. a) SSIM, b) PSNR.

**Figure 7:**
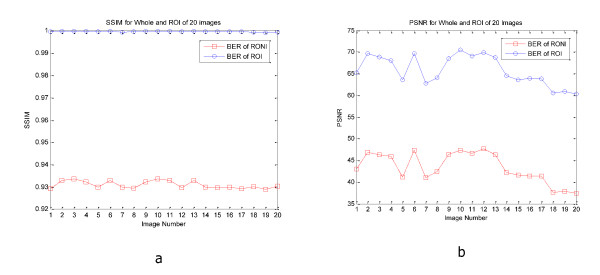
**PSNR and SSIM of whole and ROI of 20 MRI image**s. a) SSIM, b) PSNR.

**Figure 8:**
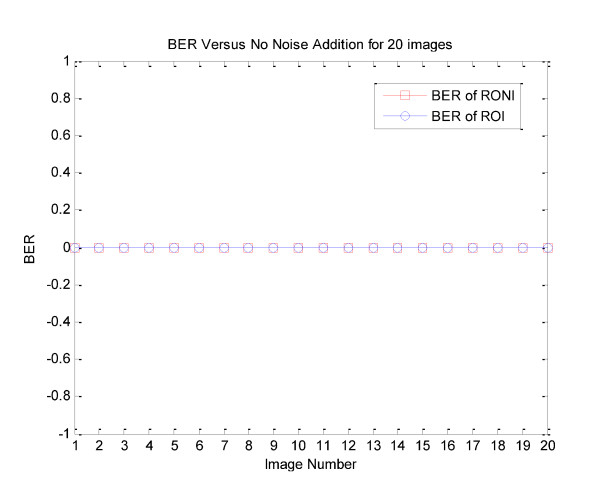
**BER of recovered signature and caption watermark versus no noise addition on the two types of image (CT and MRI)**.

**Figure 9:**
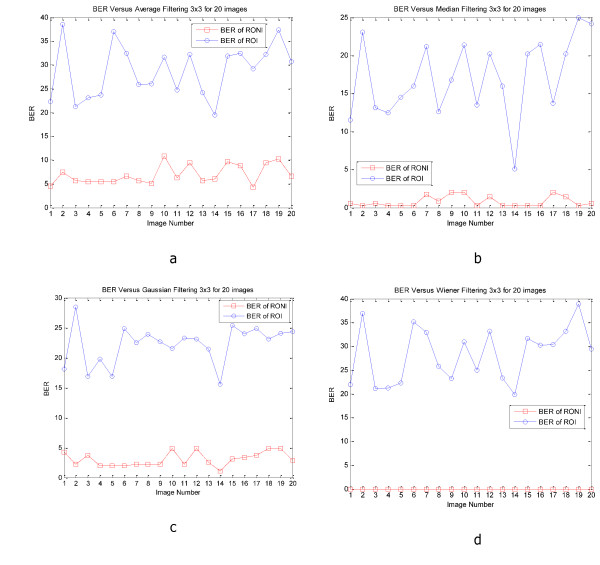
**BER of recovered signature and caption watermark various lowpass filtering on the 20 watermarked CT images**. a) Average filtering, b) Median filtering, c) Gaussian filtering, d) Wiener filtering.

**Figure 10:**
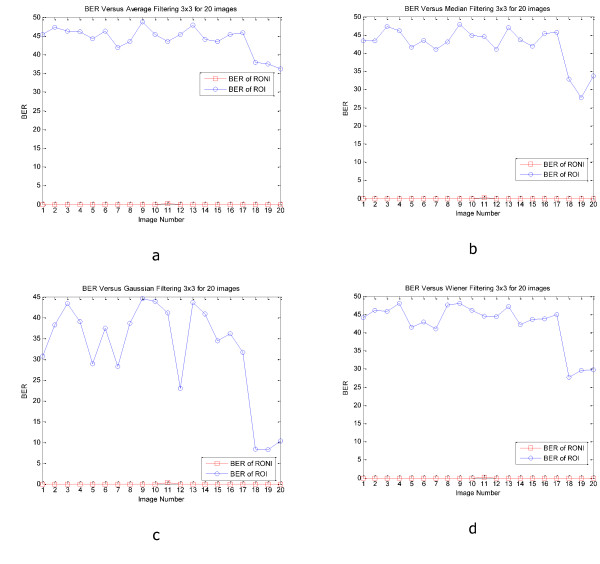
**BER of recovered signature and caption watermark various lowpass filtering on the 20 watermarked MRI images**. a) Average filtering, b) Median filtering, c) Gaussian filtering, d) Wiener filtering.

Figure 6 and 7 show PSNR and SSIM of whole and ROI of 20 CT and MRI images respectively. In these figures we can see the values of PSNR and SSIM of ROI images are respectively larger than 60 and 0.997 for CT and MRI images. These figures confirm that our algorithm has good transparency for different types of medical images. Figure 8 shows when the watermarked image is not attacked; the whole data (in ROI and RONI) is extracted correctly. Figure 9 and 10 show the results of various lowpass filtering on the two types of watermarked images (CT and MRI) in contourlet domain respectively. Because of different embedding strength, for two types of images (CT and MRI) BER values of *signature watermark *are less than BER values of *caption watermark*.

**Figure 11:**
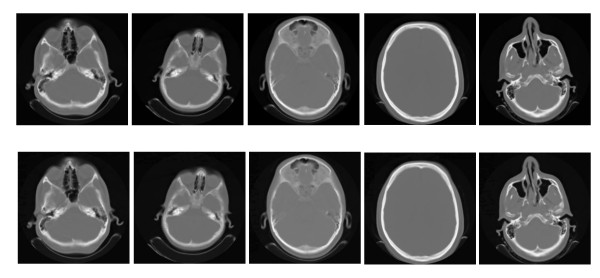
**The original (top row) and watermarked image (bottom row) in contourlet domain for several CT scans**.

To understand the effect of transform domain on our method, we select five CT images of that collection and perform the method on those in two domains contourlet and wavelet (decompose the host Image into two levels by means of Daubechies wavelet transform). For ROI watermarking in wavelet domain the basic quantization step are set to 8 and 6 for CT and MRI images respectively. For RONI embedding in wavelet domain, the basic quantization step achieved by lots of experiments are 260 and 202 for CT and MRI images respectively. Figure 11 shows these images (original) and watermarked images (in contourlet domain) together. This figure shows our method has good invisibility.

Table 1 simultaneity compares whole image and ROI in terms of PSNR and SSIM to evaluate the performance of our method in contourlet and wavelet domain. We can see that the values of PSNRs and SSIMs of ROI in contourlet and wavelet domains are respectively larger than 64 and 0.997. These values confirm that our algorithm in both domains has good transparency.

**Table 1:** A comparison between watermarked and original images in terms of PSNR and SSIM (for both whole image and ROI).

		Wavele Domain		Contourlet Domain	
	
	Region of Image	PSNR	SSIM	PSNR	SSIM
**Image 1**	**Whole Image**	46.4048	0.9435	45.9788	0.9390
	
	**ROI**	67.2762	0.9989	67.8702	0.9988

**Image 2**	**Whole Image**	46.9069	0.9408	46.6576	0.9308
	
	**ROI**	66.4100	0.9978	67.2339	0.9980

**Image 3**	**Whole Image**	45.5991	0.9395	45.0714	0.9334
	
	**ROI**	66.1855	0.9986	67.3513	0.9988

**Image 4**	**Whole Image**	46.1614	0.9411	46.1807	0.9353
	
	**ROI**	67.1661	0.9973	64.8483	0.9971

**Image5**	**Whole Image**	46.0466	0.9383	46.2871	0.9343
	
	**ROI**	66.3350	0.9987	66.9405	0.9981

**Figure 12:**
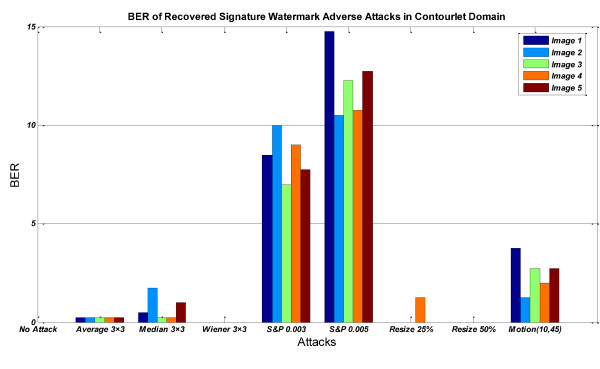
**BER of recovered *signature watermark *versus various attacks in contourlet domain**.

**Figure 13:**
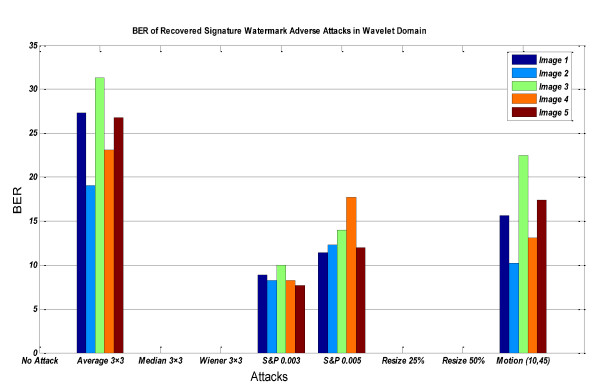
**BER of recovered *signature watermark *versus various attacks in wavelet domain**.

**Figure 14:**
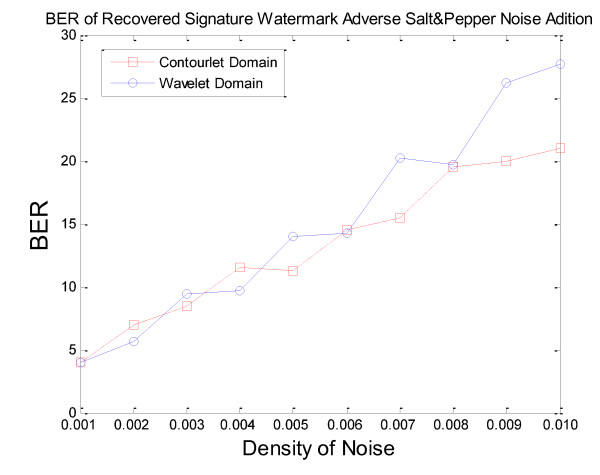
**BER of recovered *signature watermark *adverse attacks in contourlet and wavelet domain for salt&pepper noise (that based on Figures 12-13 is worst case for contourlet domain and for other types of noise contourlet-based method is very better than wavelet-based method)**.

In Figures 12, 13 &14, the BER of recovered signature watermark versus various attacks including lowpass filtering, noise addition, and resizing attacks on the watermarked image in contourlet and wavelet domain have been shown. These figures show that watermarked images in contourlet domain have greater robustness against attacks than wavelet domain and prove priority of method in contourlet domain than wavelet domain.

Table 2 shows the results of various attacks including lowpass filtering, noise addition, and resizing attacks on the watermarked image (Figure 3) in contourlet and wavelet domain.

**Table 2:** Results of various attacks on the watermarked image (Figure 3).

	Wavelet Domain			Contourlet Domain		
	***Signature***		***caption***	***Signature***		***caption***

**Attack**	**NC**	**BER**	**BER**	**NC**	**BER**	**BER**

**No Attack**	1	0	0	1	0	0

**Average Filter3 × 3**	0.7960	27.35	34.72	0.9983	0.25	37.08

**Median Filter 3 × 3**	1	0	20.18	0.9966	0.50	25.15

**Wiener Filter3 × 3**	1	0	32.85	1	0	36.39

**Salt & pepper Noise(0.003)**	0.9211	11.11	10.06	0.9485	7.50	32.73

**Salt & pepper Noise(0.005)**	0.9028	13.67	13.72	0.9236	11.00	44.03

**Resize 25%**	1	0	42.60	1	0	43.29

**Resize 50%**	1	0	20.86	1	0	25.71

**Motion (10,45)**	0.8856	15.66	43.97	0.9745	3.75	45.77

Because of different embedding strength, BER values of signature watermark are less than BER values of caption watermark. Values in Table 2 show that watermarked images in contourlet domain have greater robustness against attacks than wavelet domain. The results presented in Table 2 also show that the BER of the caption watermark is better in the wavelet domain particularly for salt and pepper noise attack. The main reason is that for ROI watermarking in contourlet domain the basic quantization step are set to 6 and 4 for CT and MRI images respectively, while for ROI watermarking in wavelet domain the basic quantization step are set to 8 and 6 for CT and MRI images respectively. The reason for these selections is balancing in transparency in ROI and RONI. As a result of these selections, robustness of caption watermark in wavelet domain would be better.

Based on the proposed domain, image (and size of image), payload, etc., the transparency and robustness of one method differs from the others. For example, we compare our results with proposed methods in [8,39,43]. The embedding capacity, PSNR, and SSIM (in ROI) for both 512 × 512 CT and MRI images in our method are respectively 0.0077 bpp, 60dB and 0.997, while these metrics for 440 × 888 CT images in [8] are 0.5168 bpp, 50.7038 dB and 0.9876 and for 512 × 512 MRI images are 0.5238 bpp, 41.2629 dB and 0.9558. For [44], the results of SSIM are not available but for 512 × 512 CT and MRI images PSNRs in dB are respectively 46.47 and 46.37. In this method the total number of embedded bits (for both modalities) are 5358 while in our method, this number is 2010. Similarly, the PSNR of 512 × 512 CT images in proposed method in [43] is 52.2 dB and the total number of embedded bits is 70799. These results show although the PSNR and SSIM indexes of our method are better (and results in high transparency) but other methods could have higher capacity.

## Conclusion

This paper presents a new watermarking method for DICOM images (various types) based on using different embedding strength for ROI and RONI in order to not affect the interpretation by medical specialists. The algorithm use an automatically selection for ROI and embed the watermark in the singular values of contourlet subbands that makes the algorithm more efficient, and robust against noise attacks than other transform domains. The embedded watermark bits can be extracted without the original image, the proposed method has high PSNR and SSIM, and the watermarked image can still conform to the DICOM format. In this paper we only tested our algorithm on CT and MR images. The approach also should be tested using other types of medical images. In addition, we achieve the embed process in the lowpass subband to improve the robustness and invisibility. However, the invisibility may be enhanced if the embed process is done in the detail subbands [44].

## Competing interests

The authors disclose that there is no "conflict of interest" including any financial and personal relationships with other people or organizations that could inappropriately influence (bias) their work.

## Authors' contributions

FR carried out gathering data, analysis and implementation. HR contributed to the study, design, analysis and testing of the results. Both authors read and approved the submitted manuscript.
